# An Ingenious Design of a High Performance-Low Complexity Image Compressor for Wireless Capsule Endoscopy

**DOI:** 10.3390/s20061617

**Published:** 2020-03-14

**Authors:** Ioannis Intzes, Hongying Meng, John Cosmas

**Affiliations:** Department of Electronic and Computer Engineering, Brunel University, London UB8 3PH, UK; Hongying.Meng@brunel.ac.uk (H.M.); John.Cosmas@brunel.ac.uk (J.C.)

**Keywords:** wireless capsule endoscopy, lossless image compression, FPGA, cadence, RTL, encounter, huffman, DPCM, multiplier-less, low-power consumption, low-complexity, subtraction, ASIC, FinFet

## Abstract

Wireless Capsule Endoscopy is a state-of-the-art technology for medical diagnoses of gastrointestinal diseases. The amount of data produced by an endoscopic capsule camera is huge. These vast amounts of data are not practical to be saved internally due to power consumption and the available size. So, this data must be transmitted wirelessly outside the human body for further processing. The data should be compressed and transmitted efficiently in the domain of power consumption. In this paper, a new approach in the design and implementation of a low complexity, multiplier-less compression algorithm is proposed. Statistical analysis of capsule endoscopy images improved the performance of traditional lossless techniques, like Huffman coding and DPCM coding. Furthermore the Huffman implementation based on simple logic gates and without the use of memory tables increases more the speed and reduce the power consumption of the proposed system. Further analysis and comparison with existing state-of-the-art methods proved that the proposed method has better performance.

## 1. Introduction

**Figure 1:**
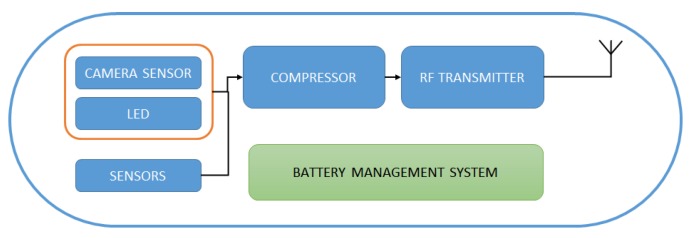
Simple block diagram of an entire endoscopic capsule system.

For many years, doctors, in order to investigate diseases of the colon, have used classical colonoscopy tools. Such tools are painful for the patients and the investigation area is limited only to the area of the colon. Wireless capsule Endoscopy (WCE) is a state-of-the-art technology for medical diagnoses of gastrointestinal diseases and illnesses [1]. The patient just swallows the capsule and the capsule does the rest of the work. A block diagram of a simple wireless endoscopic capsule is shown in Figure 1. The idea was originally conceived in 1950 [1,2], and since then a plethora of research effort has been done to improve the diagnostical procedures using new technologies. Through the years, WCE has been referred to different names like smart-pill, wireless endoscopy, video capsule, etc.

Current commercial wireless capsules utilize a resolution of up to 512 × 512 pixels and can capture and transmit up to 2 images per second [3]. In this work, a lossless image compression algorithm for WCE application is proposed. The aim is to design a low complexity, low power consumption and high compression ratio algorithm, that can improve the capsule endoscopy diagnosis. The factors taken under consideration are the power consumption, the size of the system in die and the compression ratio.

WCE are state-of-the-art electronic devices that consist of a camera sensor, lens, power unit, compression controller and wireless transmitter. Camera sensors are getting higher resolution and lower size. A huge amount of data needs to be processed and transmitted. Low complexity and high compression schemes must be used to increase the efficiency of such systems. Until now, a low resolution system exists which possess is a problem for the doctors/physicians to extract valuable results from these images. There have been a lot of works reported in image compression for WCE. Khan et al. [4], presented a lossless compression algorithm based in predictive coding. Colour conversion is used and the error produced from the conversion is encoded by Golomb-rice encoder and Unary coding. This system can compress 2 frames per second with a resolution of 320 × 240 × 24 bits. The maximum working frequency of the system is 42 MHz and the resources used are 618 cells and 2 k logic gates. Peak signal-to-noise ratio (PSNR) of the reconstructed image is *∞* because it is lossless. The average compression ratio (CR) obtained is 2.2:1. However, this system is able to compress at maximum a resolution of 320 × 240 pixels. Our system can compress up to 512 × 512 pixels. Chen et al. [5], have designed a low power compressor based on the JPEG-LS. This implementation is complex and hard to implement. The result of this system is a compressor which can process VGA resolution images. The working frequency of this system is 40 MHz and the average CR is 2.5:1. The PSNR of the reconstructed image is ∞. This implementation needs 110 k logic gates, which are too many resources in total. Despite the use of such many resources the maximum resolution that can compress is only VGA. The bottleneck in this implementation is the huge amount of hardware resources needed to work. Liu et al. [6], has implemented an image compression module for WCE that can compress at resolution of up-to 400 × 400 pixel and can have a compression ration of 3.69. Although, the bottleneck of this implementation is that the reconstructed image is not identical with the original. The PSNR of this implementation is 46.2 dB. Furthermore, this implementation needs a buffer to operate of a size of 100 Kb. Memory implementation needs area and consumes energy to operate. That’s a huge bottleneck of such type of applications. Lin et al. [7] has designed and implemented an image compression scheme based on 2D-DCT. His application is oriented in WCE usage. Although, his implementation has some bottleneck. The usage of 2D-DCT means that there is some computational cost for his implementation. Performing multiplications, divisions, data synchronization, buffering, etc., results in more area occupation and higher power consumption than systems that are not so complicated. Moreover, this implementation is not lossless, but is near-lossless and has an image quality of 32.51 dB. These implementations are not preferable due to their complexity and the loss of data. The only advantage in these techniques is the higher CR than the lossless techniques.

The rest of the paper is organized as follows. In Section 2, design criterion for the proposed image algorithm are set. Analysis of endoscopic image data is shown and the proposed compression algorithm is evaluated in software. In Section 3, FPGA design and implementation is shown and the hardware design of a low complexity, lossless compression algorithm is introduced. In Section 4, the ASIC implementation and the discussion of the hardware cost reduction with the lower power consumption is shown. Section 5 shows the conclusions and ideas for future works.

## 2. Design and Evaluation

In image compression, the main objective is to reduce the number of bits needed to represent the original image. The reason that we can compress an image is the redundancies of the image. There are some types of commonly used techniques, which are the statistical compression, spatial compression and quantizing compression. In lossless applications like this, there are some coding schemes used like Huffman coding, Run Length Encoding (RLE), Arithmetic coding, Predictive coding and Lempel–Ziv–Welch (LZV) coding [8].

The idea is to design a novel compression system which can process the image with fewer hardware resources than other systems and perform better in CR manner for the endoscopic images.

### 2.1. Endoscopic Image Dataset

A big database of capsule endoscopy images was compiled and over 200 images were used from [9,10]. These are capsule endoscopy images of various sizes. The resolution of these images varies from 150 × 150 × 24 bits, 250 × 250 × 24 bits, 512 × 512 × 24 bits, up to 1280 × 1080 × 24 bits. These endoscopic images were not only from capsule endoscopy systems but there are also from traditional endoscopy systems. That is the reason why we had some images with resolution up to 1280 × 1080 pixels. These images were used for the evaluation of our compression algorithms. Other systems [4,11,12] and [13] used these images in their experiments. So, the results could be able to be compared.

These images were from the beginning of the capsule’s travel from mouth until the end of the colon. In total, 100 from healthy persons and the rest were from patients with the most common diseases found with capsule endoscopic systems, like some types of cancer [14], blooding [15], colitis, Crohn’s disease [16], etc.

### 2.2. Proposed Image Compressor Architecture

**Figure 2:**
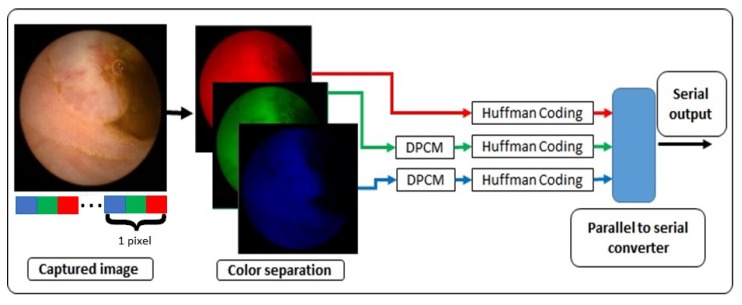
Image compressor structure.

The system is targeting high resolution RGB colour images. The proposed lossless compression architecture is shown in Figure 2. In this design, Huffman coding and Differential Pulse-Code Modulation (DPCM) are used based on the statistical analysis below. Similarities in colour range values and pixel sequences with the same or near the same values were observed. This characteristic gave us the motivation to examine the use of combinations of simple compression techniques and propose a compression algorithm dedicated for capsule endoscopy systems.

### 2.3. Statistical Analysis

In order to choose the best compression methods, statistical analysis has been done on every image and for every colour space of every pixel. The colour-space of the original images is RGB. MATLAB software was used for the experiments and the design of the compression algorithm.

**Figure 3:**
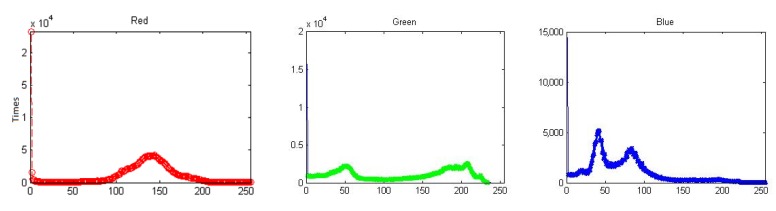
Average histogram of RGB pixel value distribution of 200 capsule endoscopy images.

**Figure 4:**
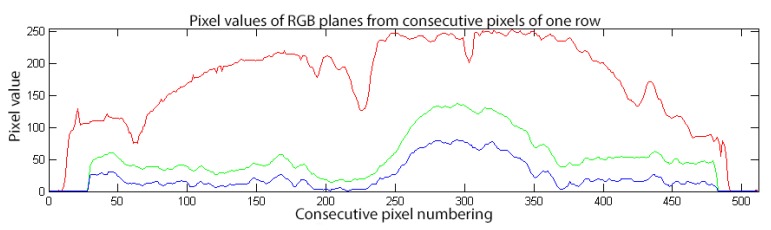
The RGB values of the pixel sequence on a single row of one capsule endoscopy image. It can be seen that Green and Blue components are much smoother than the Red component.

In Figure 3, the average distribution of all RGB images is presented. In other works, they prefer to apply colour-space transformation, like Khan et al. [4]. However, colour-space transformation might produce some estimation that is not good for lossless image compression. Huffman encoding is based in the probabilities of the pixel values. The pixels that appear more are addressed with fewer bits in the Huffman encoder output. The statistics from all the images were used to create a dedicated Huffman code book for the Red plane. The reason why we used Huffman encoding only in the Red plane and Huffman with DPCM in the rest two colours is explained clearly in Figure 4.

In Figure 4, we can see three plots of each colour plane. To understand further, due to the zig-zag scanning method that commercial camera sensor used, we plot only one line of image data. From this plot, we see that in Red colour plane the consecutive pixel values have a lot of peaks and valleys. Due to this fluctuation, applying DPCM coding to this plane will not produce the same results like in the other two colour planes. Observing the other two colour planes, it is obvious that the consecutive pixels have the same or almost the same values. Due to this, the performance of the applied DPCM encoder to these colour planes is more effective.

**Figure 5:**
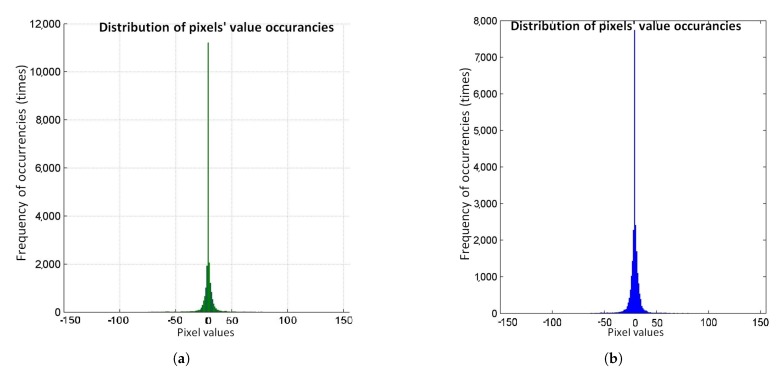
Green and Blue planes statistics after DPCM coding. (**a**) Statistical distribution of colour values for Green colour plane; (**b**) Statistical distribution of colour values for Blue colour plane.

Table 1 is showing the data output of the two DPCM encoders. For both Blue and Green colour planes. The test image is an RGB colour space image and its size is 150 × 150 pixels. The depth of every pixel is 8-bits. Figure 5 shows these data plotted. Although, from Table 1, it is easier to understand the distribution of the DPCM output. We observe that both Green and Blue planes have the most occurrences in the value of “0”. That’s the reason why in Table 2, the designed Huffman code book creates an output for input of “0” with the lowest length.

Although, in Green and Blue planes was observed that there was a correlation between neighboring pixels. Due to the repetition of the values and the fact that the neighbor pixel’s values are close, DPCM coding was applied and the distribution for Green and Blue planes are shown in Figure 5. Before DPCM code applied, a statistical analysis showed that, the pixel that repeated the most was about 10% of the total pixels in the image. After DPCM, this value is increased by up to 50%. After processing Green and Blue plane, Huffman coding was used.

### 2.4. Image Compression Algorithms

#### 2.4.1. Huffman Coding

Huffman encoding is an entropy coding used in lossless compression schemes. In Huffman coding, the pixel value which appears more frequently is represented with fewer symbols/bits. The performance of this method can be calculated using entropy. Entropy measures the amount of information presented in the data or the data randomness [17]. The average bits per pixel needed for the Huffman coding to represent the original data is given by the Equation (1).

(1) $$A=\sum_{i=1}^{M}P_{i}\times N_{i}$$

where $P_{i}$ are the probabilities of the pixels value and $N_{i}$ is the number of bits that Huffman encoder had generated.

#### 2.4.2. DPCM

In DPCM coding, the difference between the neighbor pixel values is computed. In this way, the original value pixel is decreased because of the difference of any two successive pixels is small.

In the proposed compression algorithm two different Huffman trees were used. The first one was used to compress the Red plane of the image. To produce this Huffman tree, the capsule endoscopy data-set images were used. Probabilities of every red colour value were computed and then the Huffman tree was produced. In the same way, we used the capsule endoscopy data-set images and for Green and Blue colour planes, where DPCM was applied. The output of DPCM encoder was used to produce the probabilities needed to produce the Huffman tree. In the beginning, two separate Huffman trees were produced for each colour plane, Green and Blue. It was observed that the results of the probabilities were almost the same and finally the same Huffman tree was used for both colours. In Table 2, we can see a small part of the Huffman table of Green and Blue planes and a part of the Huffman table of the Red colour plane. The input for Green and Blue planes Huffman table has negative numbers, the reason is that the input of this Huffman table is the output of the DPCM encoder, for both colours. So, the Huffman code book is designed to work with such inputs. In this way, an extra Huffman tree was not used. Green and Blue planes’ Huffman tree produces 2-bit output for the most repeated colour value and 29 bits for the less found while in Red colour plane the most repeated pixel value is reproduced with 3-bits and the less found with 15 bits. Following Equation (1), we found that for the Red plane the average bits needed to reproduce the original data is 6.85 bits/pixels. Although, in Green and Blue planes the average bits per pixel are 3.65 bits/pixel.

Due to the nature of the DPCM and Huffman coding scheme, the results are reversible. So, we cover the lossless part of the application. The second part and most important is the CR, which in our scheme is 2.2:1 on average.

### 2.5. Performance Evaluation

**Figure 6:**
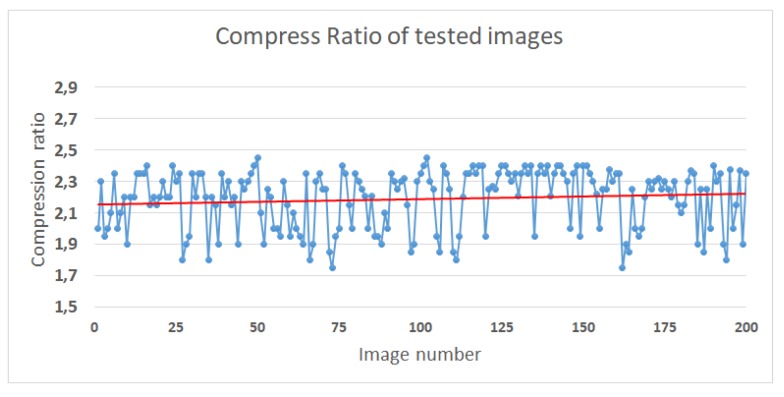
Compress Ratio performance of 200 test images. The red coloured line is the average.

In Figure 6 the performance of the proposed algorithm is shown. The average performance of the compression algorithm is 2.2:1 CR. It is obvious that 2:2 compression ratio is not achievable for all the images and some images have a compress ratio of 1.8:1 and some others perform better, about 2.45:1.

To conclude for this compression algorithm we investigate some other type of image compression. We evaluate these techniques in software to examine their performance. In Table 3, two more approaches of image compression are shown. In the first, we applied Exclusive-Or operation in the input data. This operation was made in every bit-plane for all the three colour planes (RGB). X-Or operation applied for each bit against the previous. This is was the transform coding used to prepare the data for the next stage which is the compression. For compression, Run Length Encoding (RLE) and Huffman encoding was used. The modified data by the transform encoder passed through the RLE encoder and the output imported into Huffman encoder. However, the results were not satisfactory. A lossless compression algorithm was produced but with a compress ratio that is to low. The next algorithm designed and evaluated was based in DPCM and Huffman coding. As we can see this compression algorithm is near-lossless. The reason for this is for reduction of redundancies colour-space transformation was used. The input image is in RGB format and it is transformed in YEF. In the Equation (2) the mathematical expressions to convert RGB image to YEF, are shown.

(2) $$\begin{array}{c} Y=\frac{R}{4}+\frac{G}{2}+\frac{B}{4}\\ E=\frac{R}{8}+\frac{G}{8}+\frac{B}{4}+128\\ F=\frac{R}{8}+\frac{G}{8}-\frac{B}{4}+128 \end{array}$$

As divisions are used to perform the color-space transformation. For every division performed, there are produced usually remainders. In this compression algorithm, only the integer part of the result is compressed. The remainder is not used. This is the reason why this implementation is lossy. In the inverse operation (de-compression), the result is not equal to the original image because of not compressing the remainder of the divisions.

The proposed algorithm is designed to run inside of an endoscopic capsule that is intended to be used in unhealthy intestines. Although, after statistical analysis of multiple images, it is worth performing better in healthy areas that in unhealthy, because the frames that are going to be captured are only few in the entire intestine. So, there is no degradation in the performance of our system. Furthermore, the repeatability of the same values that successive pixels have close values, using DPCM encoder we decrease the entropy and then Huffman encoder compresses the image. This characteristic is seen in the blue and green colour plane and we see it in Figure 2.

## 3. FPGA Design and Implementation

In hardware implementation, the proposed algorithm was designed and tested on a development board with a Xilinx Spartan-6 FPGA chip on it. In this implementation, as mentioned, there is a code book for Huffman coding of the red plane and a second Huffman code book for both green and blue colour planes. The code books selected to be implemented with the use of simple digital logic and not with LUT-tables and SRAM, because both of them will increase the complexity, the power consumption and the resources for the implementation.

**Figure 7:**
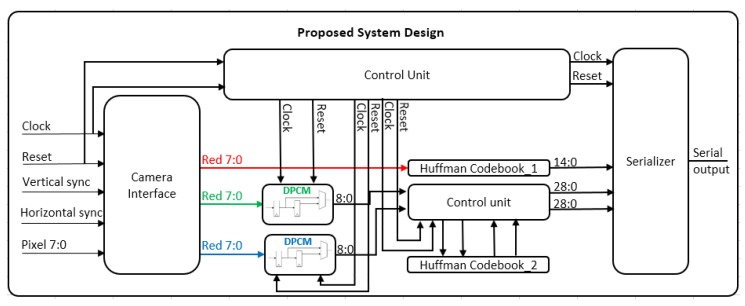
Multiplier-less hardware implementation block diagram.

In our design, we have implemented two Huffman code books, one for the Red plane and the second one for the Green and Blue plane. The Huffman code book for the red plane, compresses directly each pixel and sends the output to the serializer, although in the other two colour planes, we first apply DPCM encoder in each colour value and in the output of each DPCM encoder we apply the second Huffman code book. Both Huffman code books were produced by the use of probabilities extracted by the large capsule endoscopy image data-set. Figure 7 is the block diagram of the entire system. In the left size of the figure is the system that handles the incoming data from camera sensor. In the middle of this figure the core of the compression module is shown, with the Huffman tables and the DPCM modules. Furthermore, in the top of the figure is the Control Unit which controls the entire compression module. In the right of this figure is shown the parallel to serial converter. The DPCM output of both colour, Green and Blue, planes produces both positive and negative numbers. After processing of all the capsule endoscopy images, we found that the range of the output of the DPCM encoder was from -127 and up to 128. In our implementation we propose a Huffman tree design without the use of memory, just by using logic gates. In Huffman encoding other implementations, store the output values in a memory and each input addresses the corresponding output. In this implementation the output of the Huffman encoder is ranged from 2 to 29 bits. We solved a truth table that has 29 output functions. Each of them was computed separately. Then there were combined, and we produced a Huffman encoder without the use of memory and lookup-tables. As we said the output of the Huffman code book for Green and Blue colour planes have a maximum length of 29 bits, its output is a function of a truth table of 8-bit wide input. We solved every function separated from the others and we had 29 functions in the end.

In Equation (3) the Boolean expression of one function out of 29 is shown.

(3) $$\begin{array}{c} F0=A^{\prime }B^{\prime }C^{\prime }E^{\prime }F^{\prime }G+C^{\prime }DE^{\prime }F^{\prime }GH^{\prime }+ABC^{\prime }D^{\prime }EH+ABC^{\prime }EF^{\prime }G+A^{\prime }B^{\prime }C^{\prime }D^{\prime }F^{\prime }G^{\prime }H^{\prime }+\\ A^{\prime }B^{\prime }C^{\prime }D^{\prime }E^{\prime }GH+A^{\prime }B^{\prime }C^{\prime }D^{\prime }F^{\prime }GH+A^{\prime }B^{\prime }C^{\prime }DE^{\prime }GH^{\prime }+A^{\prime }B^{\prime }CD^{\prime }EF^{\prime }H+A^{\prime }B^{\prime }CEF^{\prime }GH+\\ BC^{\prime }DEF^{\prime }GH+BCD^{\prime }E^{\prime }F^{\prime }GH^{\prime }+A^{\prime }BCD^{\prime }FGH+A^{\prime }BCE^{\prime }FGH+A^{\prime }BCD^{\prime }EFH^{\prime }+A^{\prime }BCEFGH^{\prime }+\\ A^{\prime }BCDF^{\prime }G^{\prime }H^{\prime }+A^{\prime }BCDE^{\prime }FH^{\prime }+AB^{\prime }C^{\prime }E^{\prime }F^{\prime }G^{\prime }H^{\prime }+AC^{\prime }D^{\prime }EF^{\prime }G^{\prime }H+AB^{\prime }C^{\prime }DFG^{\prime }H^{\prime }+\\ AB^{\prime }C^{\prime }DE^{\prime }FH+ACD^{\prime }E^{\prime }F^{\prime }GH^{\prime }+AB^{\prime }CD^{\prime }E^{\prime }FH+ACD^{\prime }EF^{\prime }GH+ABC^{\prime }D^{\prime }E^{\prime }FG+ABC^{\prime }D^{\prime }EFG^{\prime }+\\ ABC^{\prime }EFG^{\prime }H+ABC^{\prime }DE^{\prime }F^{\prime }H+ABC^{\prime }DEGH^{\prime }+A^{\prime }B^{\prime }C^{\prime }D^{\prime }EFGH^{\prime }+A^{\prime }B^{\prime }C^{\prime }DEF^{\prime }G^{\prime }H+\\ A^{\prime }B^{\prime }CDE^{\prime }F^{\prime }G^{\prime }H+A^{\prime }BC^{\prime }DE^{\prime }FG^{\prime }H+A^{\prime }BC^{\prime }DEFG^{\prime }H^{\prime }+AB^{\prime }CD^{\prime }EF^{\prime }G^{\prime }H^{\prime } \end{array}$$

**Figure 8:**
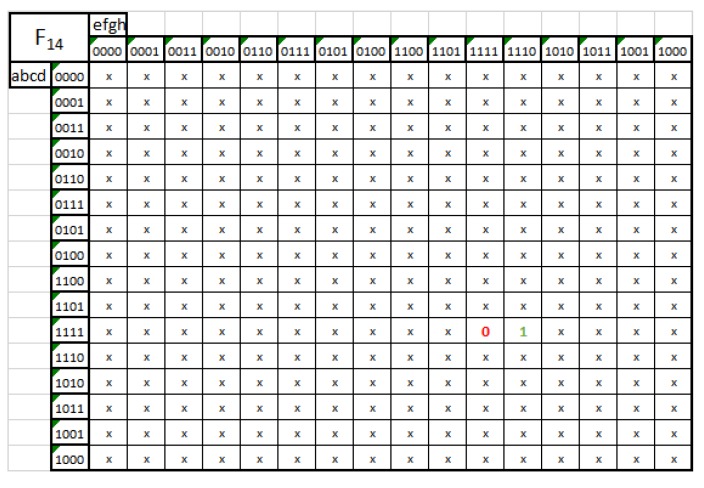
Karnaugh map for the 14th output of the Huffman encoder for Green and Blue plane.

In Equation (3), we have 8-bit input data. The input is represented by letter, from A to H. However, we can see that in this Boolean expression except of simple letters, we have letters with intonation, for example A’, this means that in our Boolean expression we use the invert input of A. Huffman table was created by the use of MATLAB software as shown in Table 4. Although, in our implementation memory elements were not used for Huffman encoder hardware implementation. So, every output of Huffman encoder was solved by the use of Karnaugh map. In Figure 8, the Karnaugh map for the 14th output of the Huffman encoder for Green and Blue plane is shown. This is the one of the 29 Karnaugh maps solved to implement the the Huffman encoder for Green and Blue planes.

Also, the use of simple logic gates circuit improves the speed of the Huffman encoder. To make the serializer able to detect the size of the output each time there is a 5-bit port for the Huffman encoder circuit, which informs the serializer the size of the output each time. Figure 7 shows the hardware structure of the proposed system. In Green and Blue planes, same Huffman code book was used. The reason to do this is the similarities of the pixel value probabilities of both colours and also for less FPGA resource usage. In this way, we used only two Huffman encoders than three. After applying DPCM encoding in both colours we need to pass the data output to the Huffman code book. In the DPCM block, we use the clock to manage the previous data input. A small FIFO has been created and is used for the DPCM operation. The size of the FIFO is 2 bytes for Green colour plane and 2 bytes for the Blue.

(4) $$\begin{array}{c} F_{10}=FH^{\prime }+DF^{\prime }G+CH \end{array}$$

(5) $$\begin{array}{c} F_{11}=F^{\prime }GH \end{array}$$

(6) $$\begin{array}{c} F_{12}=H+G \end{array}$$

(7) $$\begin{array}{c} F_{13}=G \end{array}$$

(8) $$\begin{array}{c} F_{14}=H^{\prime } \end{array}$$

The last part of this system is the serializer. In this sub-system the conversion of parallel data to serial is implemented. The data input for this system is variable. The reason is the variable output of the Huffman encoders, that can be from 2-bits and up to 29-bits for Blue and Green plane and from 3-bits to 15-bits wide for Red colour plane. Due to this variation, the parallel to serial converter needs to know the width of of the Huffman output for every processed pixel.

**Figure 9:**
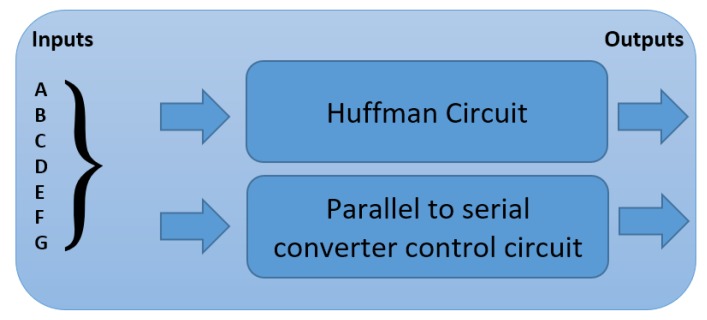
Diagram explained in graph, how the proposed Huffman encoder works and outputs extra bits to provide information to the parallel to serial converter about the width of every processed output.

As said previously, the inputs of every Huffman encoder is 8-bit wide. While the output of Huffman is variable, so there is a need to inform the parallel to serial converter, about the length of the output each time. As seen in Figure 9, if the Huffman block there is two smaller, the one is the Huffman encoder itself and the other is a circuit that exports the length of the Huffman encoder.

From Table 5, we can see the output of the circuit that outputs the size in bits of every Huffman output. For example, if the output of Huffman encoder is 10-bits wide, then this circuit outputs the value “0101”. The output range of 4-bit wide. We solved the Boolean expression for every bit. So, we get four functions, F_0_, F_1_, F_2_ and F_3_. In Equation (9). one of the four Boolean expressions is shown.

(9) $$\begin{array}{c} F2=B^{\prime }CD^{\prime }E^{\prime }F^{\prime }G^{\prime }H^{\prime }+B^{\prime }C^{\prime }D^{\prime }E^{\prime }F^{\prime }GH+B^{\prime }C^{\prime }D^{\prime }E^{\prime }FG^{\prime }+B^{\prime }C^{\prime }DE^{\prime }FG+B^{\prime }C^{\prime }DE^{\prime }FH+BCDEGH\\ +B^{\prime }C^{\prime }DEH^{\prime }+B^{\prime }C^{\prime }DEG^{\prime }+B^{\prime }C^{\prime }DEF^{\prime }+AEFG+AE^{\prime }F^{\prime }+AD^{\prime }+AC^{\prime }+AB^{\prime } \end{array}$$

In Table 6, the used FPGA resources are presented. The device is XC6SLX150T, as shown in Table 6 requires few resources from the FPGA device used. In Table 7, there is a comparison of the proposed design with other implementations. In addition, the simplicity of the algorithms requires the system to use less resources. Furthermore, because of the nature of Huffman and DPCM encoders it can be used in variable resolution images in capsule endoscopy applications.

## 4. ASIC Design and Simulation

In previous sections, a lossless, multiplier-less compression algorithm for capsule endoscopy is presented. The compression firstly was designed and tested for its performance in MATLAB software. Later the VHDL code of the proposed algorithm was produced to test and verify the performance in hardware. The last part of this work is the implementation and simulation of the compression algorithm in transistor level, ASIC design. The proposed algorithm is tested and works for an image size of 512 × 512 pixel and can work up to HD image resolution. Images used are in RGB format 8-bit colour per colour plane. For the ASIC design, of our proposed system, 16 nm FinFet technology was used. This technology combined with our multiplier-less algorithm can perform better in the domain of power. The main characteristic of FinFet technology compared with CMOS is the lower power consumption and the reduced latency in digital circuits. The use of FinFet technology can reduce the power consumption compared to CMOS up to 90 % [18].

**Figure 10:**
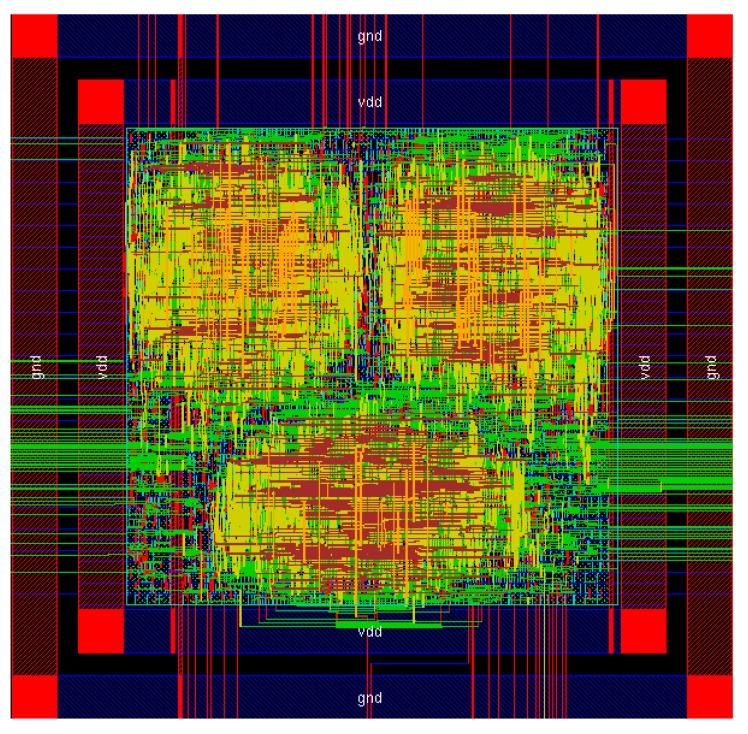
ASIC implementation of the compression algorithm by the use of Cadence software tool set.

The proposed system is designed in a way to be able to connect to any commercial image sensor that supports digital-video-port (DVP). Our compressor module is designed to have one serial output. The parallel data from the compressor are serialized and also packed with start bits and stop bits so it can directly connect to any wireless transmitter which supports serial input. The entire system was simulated and implemented by the use of Cadence Software tool-sets. In Figure 10, the final result of the proposed algorithm implemented in the transistor level is shown. In this figure the entire system is shown. As it can be seen there are three main blocks where the colour is mostly yellow. These upper two areas are the compressor itself. One block for the Red colour and the other one is the Green and Blue colours. There are almost the same size due to the use of only one Huffman table its one. The last yellow block is the parallel to serial converter and the control system to synchronize input and output data. In the left and the right of the image green lines are going out from the core. These are data lines that used for test purposes. For the design of the ASIC Cadence RTL compiler and Cadence Encounter were used. In Table 7, a comparison of our proposed compressor with existing systems is shown. Our design is a lossless, multiplier-less technique that has a CR of 2.2. The other designs are shown in Table 7, are based on near-lossless or lossy techniques that most of them are using a memory buffer to process the input image. In Table 8, a comparison of the proposed work with others is shown. In this table the proposed work is compared with others in manner of hardware cost. In more details Liu et al. [6], Gu et al. [19] and Lin et al. [7] are using input buffer for their implementation. This means more space for the IC (Integrated Circuit) implementation and more power needed to enable and keep this part of their system working. The rest of the other works presented in this table are not using input buffer memory, although, the maximum image resolution they can process is lower that our proposed compression algorithm. Our work is evaluated with input resolution of up-to 512 × 512 8-bit RGB images. A novel design of a lossless, multiplier-less and without the use of memory buffer is proposed. The proposed compressor can compress up to HD resolution images. The use of the FinFet technology and the low complexity of the compression algorithm reduces the power consumption to lower levels. In Table 9, a summary of the ASIC design of the compression is shown.

## 5. Conclusions

In this paper, a lossless compression algorithm for capsule endoscopic images has been proposed, evaluated with satisfying performance. It is further designed and implemented on a FPGA chip using only simple arithmetic operations. The ASIC was designed with Cadence tool-set by the use of 16 nm FinFet technology in 16.8k gates, without the use of any buffer memory and with a power consumption of 0.045 mW. Sample RGB endoscopy images are used to create dedicated Huffman code books. It is a low energy, low complexity, sufficient compression method which uses simple arithmetic operations. The only arithmetic operation used is subtraction and it was in the DPCM encoders of the Green and Blue planes. The output of the DPCM encoders is a signed value where we treat them as unsigned values due to their statistics by the Huffman encoder. So, in this way, we do not increase the complexity of the design. The maximum image size can be processed is up to HD resolution. With the 2.2:1 CR, we achieved the same results as other works but with less resources, low energy, higher resolution and simpler technique.

Further improvement can be done by applying a clipping algorithm at [4]. Because the lens of the capsule generates a circular image and the sensor camera is rectangular, the corners of the image have a value of zero. With this cropping algorithm, the compression performance might be improved further.

## Figures and Tables

**Table 1:** Table shows a few components of and their time of occurrences of Blue and Green plane after DPCM code is applied.

Red Value	Percentages	DPCM Output	Blue Plane	Percentages	Green Plane	Percentages
0	10.7	−42	2	0.009%	1	0.005%
1	14.18	−41	2	0.009%	2	0.009%
2	2.28	−39	2	0.009%	2	0.009%
3	0.59	−38	2	0.009%	1	0.005%
4	0.55	−37	5	0.022%	3	0.013%
5	0.48	−36	1	0.005%	2	0.009%
6	0.23	−35	2	0.009%	1	0.005%
…	…	…	…	…	…	…
120	0.24	−5	429	1.9%	400	1.77%
121	0.2	−4	661	2.94%	623	2.76%
122	0.21	−3	1045	4.64%	988	4.39%
123	0.29	−2	1473	6.55%	1325	5.88%
124	0.25	−1	2322	10.32%	2655	11.8%
125	0.17	0	7386	32.83%	11655	51.8%
126	0.24	1	2451	10.89%	2835	12.6%
127	0.3	2	1737	7.72%	2014	8.95%
128	0.31	3	1126	5%	1057	4.69%
129	0.29	4	836	3.71%	923	4.1%
130	0.29	5	536	2.38%	618	2.74%
…	…	…	…	…	…	…
249	0.13	28	10	0.044%	9	0.04%
250	0.14	29	12	0.053%	12	0.053%
251	0.11	30	8	0.035%	7	0.031%
252	0.08	31	6	0.026%	7	0.031%
253	0.03	32	2	0.009%	3	0.013%
254	0.02	33	5	0.022%	2	0.009%
255	0.01	34	1	0.005%	1	0.005%

**Table 2:** Table showing a few components of the Huffman tree for Green and Blue planes.

Input	Huffman Output for Green and Blue Planes	Input	Huffman Output for Red Plane
−127	10001101111000100000111100001	0	101
−126	10001101111000100000111100011	1	010
−125	10001101111000100000111101011	2	11101
…	…	…	…
−1	101	127	11011111
0	00	128	11010111
1	100	129	11110000
…	…	…	…
127	010101101000111110	254	001000111110111
128	01010110100011110	255	001000111110110

**Table 3:** Compression performance of different algorithms in software evaluation. XOR-RLE-HUF means applying XOR logical operation in every bit plane of an 8-bit depth colour plane. Then, Run length encoding and finally Huffman is applied. DCPM-HUF means DPCM encoding is applied in every colour plane, and next Huffman encoding is applied.

Compression Method	Colour Space	Avg. CR	PSNR (dB)
XOR-RLE-HUF	RGB	1.6	*∞*
DPCM-HUF	YEF	1.9	37.9
This work	RGB	2.2	*∞*

**Table 4:** Truth table of Red plane Huffman code book.

Data Input	F_0_	F_1_	F_2_	F_3_	F_4_	…	F_10_	F_11_	F_12_	F_13_	F_14_
00000000	1	0	1	x	x	…	x	x	x	x	x
00000001	0	1	0	x	x	…	x	x	x	x	x
00000010	1	1	1	0	1	…	x	x	x	x	x
00000011	1	1	1	0	0	…	x	x	x	x	x
00000100	1	1	1	1	1	…	x	x	x	x	x
00000101	0	0	0	1	1	…	x	x	x	x	x
.											
.											
.											
11111010	1	1	0	1	1	…	x	x	x	x	x
11111011	0	0	1	0	0	…	1	1	x	x	x
11111100	0	0	1	0	0	…	1	0	0	x	x
11111101	1	0	1	x	x	…	1	0	1	0	x
11111110	0	1	0	x	x	…	1	0	1	1	1
11111111	0	0	1	0	0	…	1	0	1	1	0

**Table 5:** Table of data output in correspondence of Huffman output.

Size of Huffman Output in Bits	Data Output
3	0000
5	0001
7	0010
8	0011
9	0100
10	0101
11	0110
12	0111
13	1100
14	1001
15	1010

**Table 6:** FPGA resources used.

Resources	Used/Available	Utilization
Slices	288 out of 184,304	0%
4-input LUTs	2816 out of 92,152	3%
DSP48A1s	2 out of 180	2%
Max. Frequency	144 MHz	-

**Table 7:** Comparison between proposed method and other existing methods.

Specifications	Mostafa [13]	Mostafa [12]	Li [11]	Khan [4]	Our Work
Colour plane	YUV	RGB	RGB	RGB	RGB
Compression method	Pred. Coding	DCT	JPEG-LS	Pred. Coding	Pred. Coding
Buffer	NO	YES	YES	NO	NO
lossless	YES	NO	near-lossless	YES	YES
Image size	320 × 240	-	640 × 480	320 × 240	512 × 512
Clock rate	91 MHz	150 MHz	40 MHz	42 MHz	144 MHz
Compression ratio	2.4	10	3.5	2.2	2.2
PSNR (dB)	*∞*	32.95	45	*∞*	*∞*

**Table 8:** Comparison between proposed compression scheme with others in manner of hardware cost.

	Core Size (mm${}^{2}$)	Gates	Buffer	Power (mW)	Image Size	Lossless
Mostafa et al. [13]	0.0256	2 k	0	18	320 × 240	YES
Liu et al. [6]	23.04	27.8 k	100 kb	N/A	400 × 400	NO
Gu et al. [19]	3	30 k	4.5 kb	1.03	480 × 480	NO
Lin et al. [7]	0.39	31 k	288 b	14.92	512 × 512	NO
Goyal et al. [20]	0.019	4.9 k	-	0.0142	256 × 256	NO
Fante et al. [21]	0.018	2.2 k	-	0.035	256 × 256	NO
This work	0.056	16.8 k	0	0.045	512 × 512	YES

**Table 9:** ASIC design performance.

ASIC	Feature
Technology	FinFet 16 nm
Area	0.056 mm${}^{2}$
Clock frequency	144 Mhz
Voltage	0.85 V
Logic Gate	16.8 K
Memory	0 kb
Power consumption	4.5 mW
